# Aberrant expression of PAR bZIP transcription factors is associated with epileptogenesis, focus on hepatic leukemia factor

**DOI:** 10.1038/s41598-020-60638-7

**Published:** 2020-02-28

**Authors:** Lukas Rambousek, Tilo Gschwind, Carlos Lafourcade, Jean-Charles Paterna, Linda Dib, Jean-Marc Fritschy, Adriano Fontana

**Affiliations:** 1 Institute of Experimental Immunology, Winterthurerstrasse 190, University of Zurich, 8057 Zurich, Switzerland; 2 Institute of Pharmacology and Toxicology, Winterthurerstrasse 190, University of Zurich, 8057 Zurich, Switzerland; 3 Neuroscience Center Zurich, University of Zurich and ETH Zurich, 8057 Zurich, Switzerland; 4 Laboratorio de Neurociencias, Universidad de los Andes, 7620157 Santiago, Chile; 5 Viral Vector Facility, Neuroscience Center Zurich, University of Zurich and ETH Zurich, 8057 Zurich, Switzerland; 6 Swiss Institute of Bioinformatics, 1015 Lausanne, Switzerland

**Keywords:** Circadian rhythms and sleep, Diseases of the nervous system, Neuroimmunology, Neurology, Epilepsy

## Abstract

Epilepsy is a widespread neurological disease characterized by abnormal neuronal activity resulting in recurrent seizures. There is mounting evidence that a circadian system disruption, involving clock genes and their downstream transcriptional regulators, is associated with epilepsy. In this study, we characterized the hippocampal expression of clock genes and PAR bZIP transcription factors (TFs) in a mouse model of temporal lobe epilepsy induced by intrahippocampal injection of kainic acid (KA). The expression of PAR bZIP TFs was significantly altered following KA injection as well as in other rodent models of acquired epilepsy. Although the PAR bZIP TFs are regulated by proinflammatory cytokines in peripheral tissues, we discovered that the regulation of their expression is inflammation-independent in hippocampal tissue and rather mediated by clock genes and hyperexcitability. Furthermore, we report that hepatic leukemia factor (*Hlf*), a member of PAR bZIP TFs family, is invariably downregulated in animal models of acquired epilepsy, regulates neuronal activity *in vitro* and its overexpression in dentate gyrus neurons *in vivo* leads to altered expression of genes associated with seizures and epilepsy. Overall, our study provides further evidence of PAR bZIP TFs involvement in epileptogenesis and points to *Hlf* as the key player.

## Introduction

Epilepsy is a chronic brain disease characterized by the occurrence of recurrent seizures, which are the result of an excessive electrical discharge or hypersynchronization of a group of neurons in distinct areas of the brain. Temporal lobe epilepsy (TLE) is the most common subtype of acquired epilepsy^1^. TLE, affecting the hippocampal formation and surrounding cortices of the temporal lobe, is associated with pathological changes including neuroinflammation and neurodegeneration resulting in impaired cognition^2^.

Recent studies provided converging evidence that the circadian system might be disrupted in epilepsy^3,4^. At the molecular level, core clock genes form a transcriptional-translational feedback loop that drives their diurnal oscillations. The positive loop members CLOCK (or NPAS2) and BMAL1 form heterodimers that activate the expression of negative feedback loop members, CRY and PER, by binding to E-box motives in their promoters. CRY and PER consequently inhibit the transcriptional activity of CLOCK/NPAS2:BMAL1 complex and thereby their own expression. The inhibition of BMAL1 expression is additionally mediated in a second negative feedback loop by REV-erb-α and RORC^5,6^. Moreover, core clock components interact with the expression of the proline and acidic amino acid-rich basic leucine zipper (PAR bZIP) transcription factors (TFs). This family is composed ﻿of three activators, DBP (albumin D-site-binding protein), HLF (hepatic leukemia factor), TEF (thyrotrophic embryonic factor) and one suppressor of transcription E4 Promoter-Binding Protein 4 (E4BP4), known also as NFIL3^7–10^. It has been shown that E4BP4 has an opposite rhythm of oscillation compared to positive members of PAR bZIP TFs and compete with them for the same binding sites^11^. Importantly, the triple knockout mouse deficient for the main three PAR bZIP transcription factors (*Hlf*, *Dbp* and *Tef*) developed generalized spontaneous seizures^12^. Additionally, a significant reduction in seizure threshold was reported in *Bmal1* knockout mouse^13^ and decreased levels of CLOCK, CRY1, PER1 and DBP protein were found in epileptic tissue obtained from surgical resection^3^.

Proinflammatory cytokines, which may be produced following seizures and contribute to their generation and susceptibility^14^, have been shown to be key regulators of clock gene expression. Decreased expression of clock genes as well as PAR bZIP TFs was observed in NIH-3T3 cells or human synovial fibroblasts exposed to tumor necrosis factor α (TNF-α) or interleukin 1-β (IL-1β), and in the liver of mice injected with TNF-α or with CD40 agonistic antibodies^15–18^. The functional significance of cytokine-mediated suppression of clock genes remains elusive.

In this study, our objective was to characterize the hippocampal expression of clock genes and PAR bZIP TFs in a kainic acid (KA) model of TLE and to dissect the interaction between them and neuroinflammation. The intrahippocampal KA injection in adult mice induces an acute status epilepticus, followed by 14 days of a latent period without ictal activity. Afterwards, animals develop spontaneous recurrent seizures^19^. In this model, seizures are accompanied by progressive neurodegeneration and neuroinflammation in hippocampus^20^.

We found significant changes in PAR bZIP TFs expression characterized by downregulation of *Hlf*, *Dbp*, *Tef* and upregulation of *E4bp4*. Surprisingly, we were not able to mimic these changes by simulating only neuroinflammation. This suggests a tissue-specific and inflammation-independent pathway. The changes in *Clock*, *Bmal1*, *Npas*2 and *Period* genes expression at an early stage of KA-induced epilepsy might be driving the subsequent changes in PAR bZIP TFs expression. Additionally, we demonstrated that *Hlf* over-expression in primary hippocampal neurons leads to altered neuronal excitability *in vitro* and differential expression of genes involved in neuronal excitability, seizures and epilepsy *in vivo*.

## Methods

### Animals

All methods were carried out in accordance with guidelines and regulations of University of Zurich, Switzerland and University of Los Andes, Santiago, Chile. All experimental protocols were approved by the Cantonal Veterinary Office of Zurich, Switzerland and the Bioethical Committee of University of Los Andes, Santiago, Chile. Adult C57Bl6/J male mice (12–14 weeks old, from Charles River) and Sprague Dawley female rats (from the breeding colony of Universidad de los Andes, Chile) were used in the study. Animals were housed at standard conditions (20–24 °C, minimum 40% relative humidity) under a 12-hour light/dark cycle (lights on at 7 a.m., lights off at 7 p.m), with access to food and water *ad libitum*.

### Stereotaxic injections into hippocampus

Mice were anaesthetized with isoflurane and placed into a motorized stereotaxic robot (Neurostar) equipped with the drill and microinjector Nanoinject II (Drummond). The concentration of isoflurane was adjusted until all pain reactions disappeared. After fixation, a small opening was drilled in the skull to allow for the injection needle to access the hippocampal formation. Afterwards, sterile solutions of PBS, KA (5 nM, 70 nl, Sigma Aldrich), N-methyl-D-aspartic-acid (NMDA, 70 mM, 140 nl, Sigma Aldrich), (-)-Bicuculline methiodide (1 μM, 0.5 μl, Tocris), mouse recombinant cytokines TNF-α (15 pmol, 0.5 μl, Peprotech) and IL-1β (5 pmol, 0.5 μl, Peprotech), lipopolysaccharide (LPS, 10 μg/1 μl, 1 μl, Sigma Aldrich) or AAV vectors (described below) were stereotaxically injected into the right hippocampus (−1.8 AP, +1.6 ML, +1.9 DV). The injection capillary was left in the brain for additional 5 min after the injection. Control animals received a matching volume of sterile PBS or control AAV vector. All injections were performed between 7 a.m. and noon. The concentration and volume injected is summarized in Table 1. Additionally, before and immediately after surgery, mice received an injection of Temgesic (buprenorphine, 0.06 mg/kg, i.p.).

**Table 1:** Primers used in the RT-qPCR.

Gene Name	Forward primer	Reverse primer
*Hlf*	TGCTTCGTCGTGCGTCTC	CAAGAGGGAAATGGAGAAAGTGAA
*Tef*	TCCCCTTAGTCCCCGTTCT	CTCCAAGAAACAAGCAGACAGT
*Dbp*	TAGAAGGAGCGCCTTGAGTC	GCAACCCTCCAGTATCCAGA
*E4bp4*	ATGGGAAGCTCTTTCTCCACT	TACCCGAGGTTCCATGTTTC
*Npas2*	TCGGGACCAGTTCAATGTTCT	GATTTCTGTTTGTGCTGAGACTTC
*Clock*	TTCCTTCCTTAGAGACGAGACT	CTAAATGCTACCCTGAGGATAGAG
*Bmal1*	AACTACAAGCCAACATTTCTATCAG	TTCCCTCGGTCACATCCTAC
*Per1*	CAGGCTAACCAGGAATATTACCAGC	CACAGCCACAGAGAAGGTGTCCTGG
*Per2*	CTCAGCCTCCCACTCTATATGT	AGGACCAGCAGCACAGATAG
*Per3*	TCAGAAGAAGCCAAGCCAATC	GTTGTTTCCTGTTTCCGTATGTC
*Cry1*	GATCCACCATTTAGCCAGACAC	ACAGCCACATCCAACTTCCA
*Cry2*	CAAGCACTTGGAACGGAAGG	GAAGAGGCGGCAGGAGAG
*Rev-erb-α*	GTCTCTCCGTTGGCATGTCT	CCAAGTTCATGGCGCTCT
*Rorc*	ACTACGGGGTTATCACCTGTGAG	GTGCAGGAGTAGGCCACATTAC
*Hprt*	TCCTCCTCAGACCGCTTTT	AGGTATACAAAACAAATCTAGGTCAT
*Eef1a1*	AAGCCCATGTGTGTTGAGAG	CTCCAGCAGCCTTCTTGTC
*TNF-α*	GAAGATGTGCCTGTCCTGTGT	CGCTCAGGTCAGTGATGTTAA
*Il-1β*	AAACAGATGAAGTGTAGG	TGGAGAACACCACTTGTT
*Il-10*	CAGGGATCTTAGCTAACGGAAA	GCTCAGTGAATAAATAGAATGGGAAC

In KA model experiments (n = 125, 4–7 mice per group), animals were sacrificed at either 3 hours, 1-day post injection (dpi), 6 dpi, 14 dpi or 28 dpi. Additionally, at 1 and 28 dpi, mice were sacrificed at four different Zeitgeber time-points (ZT = Zeitgeber time, ZT0 lights on, ZT12 lights off): ZT0, ZT6, ZT12 and ZT18. At 6 and 14 dpi, mice were sacrificed at two ZT: ZT0 and ZT12. In experiments investigating acute effect of recombinant murine cytokines TNF-α and IL-1β (n = 15, 5 mice per group), animals were sacrificed at 6 hours after their injection. In experiments with LPS (n = 10, 5 mice per group), NMDA and (-)-Bicuculline methiodide (n = 15, 5 mice per group), animals were sacrificed at 24 h after the injection to resemble the 1 dpi time-point in KA experiment.

### AAV-vectors

Self-complementary (sc) adenovirus-associated virus (AAV) vector TNF (AAV-TNF) scAAV-2/8-hCMV-chI-floxedmTNFalpha-SV40p(A) and its corresponding control AAV vector (AAV-control) scAAV-2/8-hCMV-chI-loxP-SV40p(A) were kindly provided by the laboratory of Christopher Pryce (University of Zurich, Switzerland). The AAV vectors were characterized in their recent publication [21]. AAV-control and AAV-TNF were injected as described above at a final titer of 1.1 × 10^11^ vg/ml (280 μl, n = 10, 5 mice per group). Animals were sacrificed 14 days after AAV injection and their brain processed for the gene expression analysis.

**Figure 1:**
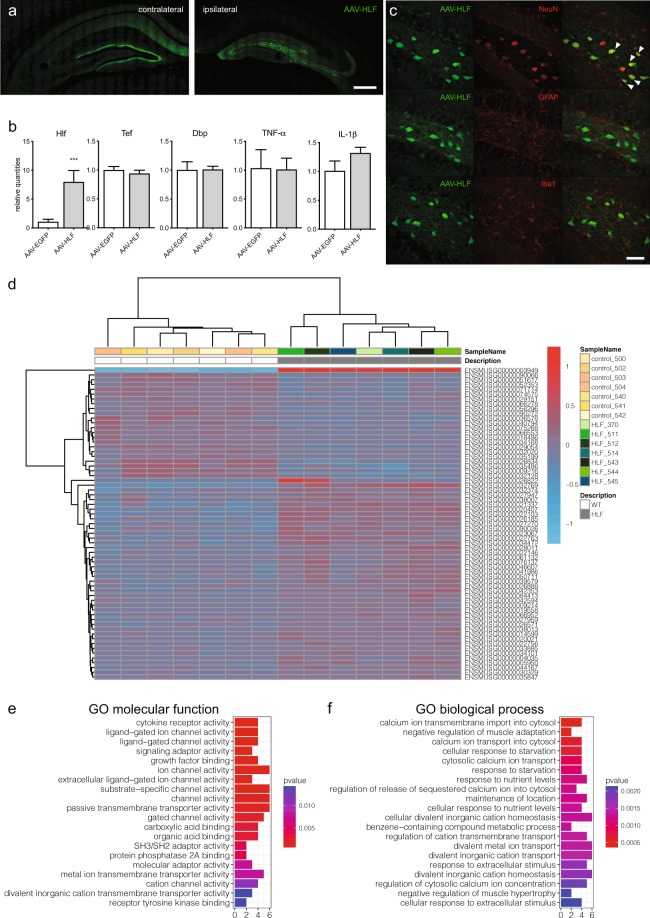
Identification of genes associated with *Hlf* over-expression in *dentate gyrus* neurons of adult male mice. (**a**) The representative expression pattern of EGFP reporter expression after AAV-HLF injection in the ipsilateral hippocampus. Scalebar = 400 μm (**b**) Relative mean expression (±SD) of PAR bZIP TFs and inflammatory cytokines after AAV-HLF injection. The t-test was performed to evaluate significance. ***p < 0.001. (**c**) Co-localization of EGFP reporter expression with immunohistological staining specific for neurons (NeuN), astrocytes (GFAP) and microglia (Iba1). White arrows show co-localization of HLF-EGFP reporter with neuronal NeuN staining. Scalebar = 50 μm. (**d**) Heatmap shows the z-score from the transformed counts of each individual sample replicate, for all differentially expressed genes. (**e**) Summary of enrichment for GO molecular function and (**f**) Biological process. Figures were created using Adobe Illustrator version 23.1.1. www.adobe.com/Illustrator, R version 3.4.3.; https://www.R-project.org and GraphPad Prism version 8.3.1 for macOS, www.graphpad.com.

Single-stranded (ss) AAV vector HLF (AAV-HLF) ssAAV-2/8-hSyn1-chI-EGFP_2A_mHLF-WPRE-SV40p(A) and its corresponding control AAV vector (AAV-EGFP) ssAAV-2/8-hSyn1-chI-EGFP-WPRE-SV40p(A) were produced by the Viral Vector Facility (VVF, University of Zurich) as described before^21^. AAV-HLF was constructed by inserting the PCR-amplified mHLF ORF (Origene, MR204073, NM_172563) downstream and in-frame of an EGFP_2A sequence. AAV-EGFP and AAV-HLF vectors were injected at a final titer of 1.2 × 10^12^ vg/ml (280 μl, n = 26, 7 mice per group for transcriptomics; 12 mice were used in the pilot study to determine the transgene expression levels, localization and subcellular specificity in the hippocampus, Fig. 1a–c). After 14 days of expression, animals were sacrificed, and their brain processed for the gene expression and fluorescent microscopy analyses. Histology and immunohistochemistry were performed as described in our previous work^22,23^. Brain sections were stained with the following antibodies: GFAP (DAKO Schweiz, Z334, 1:20000), Iba-1 (Wako, 019–19741, 1:3000) and NeuN (Chemicon, MAB377, 1:1000).

### RNA isolation and gene expression analysis

In all experiments, the dorsal parts of ipsilateral and contralateral hippocampi were dissected on ice and were immediately frozen in RNAPure PeqGOLD solution (VWR). At the day of RNA isolation, brain tissue samples were homogenized (Qiagen TissueLyser) and whole cell RNA extracted using NucleoSpin-RNA II kit (Macherey-Nagel). 500 μg RNA was subsequently reverse-transcribed using random hexamer primers (Thermo Scientific) and M-MuLV reverse transcriptase (Life Technologies). 20 ng of cDNA was amplified in a CFX384 detection system (Biorad) using PrecisionPLUS qPCR Mastermix (Primerdesign). The relative levels of each RNA sample were calculated by the 2-□ΔCT method^24^ using qbase software (Biogazelle). *Hprt1* and *eEF1a1* were used as housekeeping genes. Each CT value used for these calculations was the mean of triplicates of the same reaction. All primers used were synthetized by Eurofins genomics. Sequences of all used primers are listed in Table 1. The t-test with multiple-testing correction was performed to evaluate statistical significance in the gene expression using the function “t-test” on R statistical package^25^.

### Analysis of GSE47752 dataset

The Affymetrix microarray data described by Dingledine *et al*.^26^ was obtained from the Gene expression omnibus (GEO), under accession number GSE47752. This data is composed of 172 samples and 31099 features and was analyzed using the R statistical software^25^. The following samples were included in the analysis: kainic acid (Wadman-day 1-rat 1–6, Wadman-control-rat 1–6, Nadler-day 1-rat 1–6, Nadler-control-rat 1–6; n = 12 control, n = 12 kainic acid), pilocarpine (Coulter-day 1-rat 1–6, Coulter-control-rat 1–6, Dingledine-day 1-rat 1–6, Dingledine-control-rat 1–6; n = 12 control, n = 12 pilocarpine), kindling (McNamara-stage 2-rat 1–6, McNamara-control-rat 1–6, Rogawski-stage 2-rat 1–6, Rogawski-control-rat 1–6; n = 12 control, n = 12 kindling) and self-sustained status epilepticus (Wasterlain-day 1-rat 1–6, Wasterlain-control-rat 1–5; n = 5 control, n = 6 self-sustained status epilepticus).

Normalization was performed using the RMA normalization using all probes regardless of Affymetrix ‘Present’ (P) or ‘Absent’ (A) calls. Only probes with the “at” suffix were retained to ensure that only probes specific to a single gene were carried forward for further analysis. Differential expression of the genes *Hlf*, *Tef*, *Dbp* and *E4bp4* was performed using the Limma (Linear models for microarray) package^27^.

### Primary neuronal cultures & patch clamp

Primary hippocampal cultures were prepared from E18 Sprague Dawley rats as previously described^28^. This protocol was approved by the Universidad de los Andes Animal Care and Use Committee. Cells were cultured on cover slips and transduced with at 2 DIV with AAV-HLF or AAV-EGFP (MOI = 50000, 1.2 × 10^13^ vg/ml). Medium was changed every 3 days. At ~12–16 DIV, the coverslip was transferred to a 35 mm plate containing ACSF which contained (in mM) 120 NaCl, 3 KCl, 2.5 CaCl2, 2 MgSO4, 1 NaH2PO4, 25 NaHCO3, and 20 glucose (pH 7.4) for electrophysiology experiments. The intracellular solution consisted of (in mM) 146 KGluconate (KGluc), 1 NaCl, 1 MgSO4, 0.2 CaCl2, 2 EGTA, 10 HEPES, 4 MgATP, 0.3 NaGTP, (pH 7.3, 290 mOsM/Kg). Patch clamp experiments were performed under an inverted NIKON TE-2000U microscope to visualize transduced primary hippocampal neurons expressing EGFP. Whole-cell pipettes were pulled from thin wall glass capillaries (1.5 O.D. × 1.17 I.D., Harvard Apparatus, Massachusetts, USA) using a Sutter puller9 (model P-97). Electrode resistances in the bath were 5–7 MΩ. Series resistance was monitored by a 5 mV step, and cells were discarded if this changed significantly (~20%). Cells were clamped at a holding potential of −70 mV with an axopatch 200B (Molecular Devices, Pennsylvania, USA), filtered a 2 KHz and digitized at 5 kHz using a Digidata 1550 (Molecular Devices) and Clampex 10.0 (Molecular Devices). Stimulation of neurons was achieved by adding bicuculline (20 μM) to the recording bath, while delivering glutamate (2.5 mM) with a patch pipette with a resistance of ~2MΩ placed near the recorded cell (~50μm away). Glutamate concentration in the pipette was 2.5 mM, and delivery of glutamate was obtained by a 50 μl Hamilton syringe mounted to a SP101i syringe pump (flow rate of 1 μl/min, World Precision Instruments, Fl, USA) and connected to the pipette by a tight silicon tubing. Action potentials were obtained by applying a series of current steps (50 pA each) under current clamp mode. Traces were recorded with pClamp 10 and analysis of intrinsic properties and sPSCs were performed with MiniAnalysis (Synaptosoft). Average traces were obtained by selecting events in MiniAnalysis, aligning the peak of the events and averaging those traces under the “group analysis” function. Average traces from each cell were averaged to obtain one trace per group. Decay fit was calculated in Mini Analysis by using a 2 exponential function approach (Amplitude1 exp(−time/τ1) + Amplitude2 exp(−time/τ2).

Two-way ANOVAs and two-tailed t-tests were performed with GraphPad Prism version 8 (GraphPad Software). The amplitude and interevent interval of PSCs were analyzed according to the highest level of activity shown, in one-minute long traces. To make the KS tests more stringent we applied a bootstrap estimation of the p-value by extracting random samples of size 100 (either currents or amplitudes) using the function “sample” on R statistical package^25^. After obtaining a p-value from these samples the procedure was repeated 1000 times. The percentage of times the random samples gave a p-value < 5% was used to establish if cumulative distributions are different. We considered distributions to be significantly different when more than 50% of random samples had a p-value < 5%This ensures that the KS test is not as sensitive to small differences between the associated functions. Density plot of sPSCs amplitudes and interevent intervals were used to visually confirm our statistical results.

### Transcriptomics analysis

Next-generation sequencing was performed by Functional Genomics Center Zurich, University of Zurich, Switzerland. Consequent data analysis was than performed by the Swiss Institute of Bioinformatics, Switzerland. Total RNA was processed using the TruSeq RNA stranded protocol (Illumina) in order to produce sequencing libraries. Libraries were then sequenced on the Illumina HiSeq. 4000, single-end, 125 cycles. Reads were aligned against Mus Musculus.GRCm38.86 genome using STAR (v.2.5.2b)^29^. The number of read counts per gene locus was summarized with htseq-count (v0.6.1)^30^ using Mus Musculus.GRCm38.86 gene annotations. Quality of the RNA-seq data alignment was assessed using RSeQC (v. 2.3.7)^31^. Reads were also aligned to the Mus Musculus.GRCm38.86 transcriptome using STAR (v. 2.5.2b)^29^ and the estimation of the isoforms abundance was computed using RSEM (v. 1.2.31)^32^. To assess differential expression, we used the R Bioconductor package DESeq. 2 (version 1.14.1)^33^. Differentially expressed Genes were identified at the Benjamini-Hochberg (BH) adjusted P < 0.05 level, using the Wald test. For gene set enrichment analysis, no direction criterion on fold change was applied. Enriched GO (Gene Onthology) categories were identified using the enrichment analysis package in R/Bioconductor, clusterProfiler^34^ with significant terms at BH P adjusted <0.05 reported.

## Results

### Status epilepticus induces changes in the expression of PAR bZIP transcription factors and does not affect expression of clock core genes

**Figure 2:**
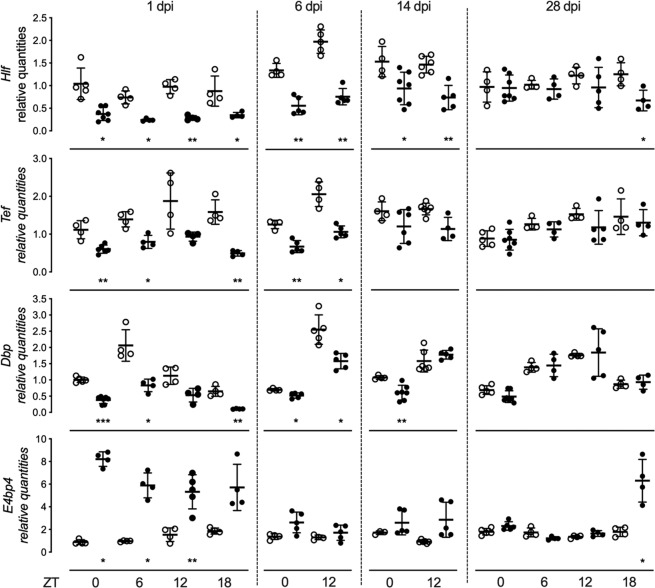
Relative mean expression (±SD) of PAR bZIP TFs (*Hlf*, *Tef*, *Dbp* and *E4bp4*) in ipsilateral dorsal hippocampus at 1, 6, 14 and 28 days post injection (dpi) of KA in adult male mice. ZT0, ZT6, ZT12 and ZT18 (ZT = Zeitgeber time, ZT0 lights on, ZT12 lights off). White and black circles represent individual values for control and KA groups, respectively. The t-test with multiple-testing correction was performed to evaluate significance between control and KA group for each gene. *p < 0.05, **p < 0.005, ***p < 0.00. Figures were created using GraphPad Prism version 8.3.1 for macOS, www.graphpad.com.

**Figure 3:**
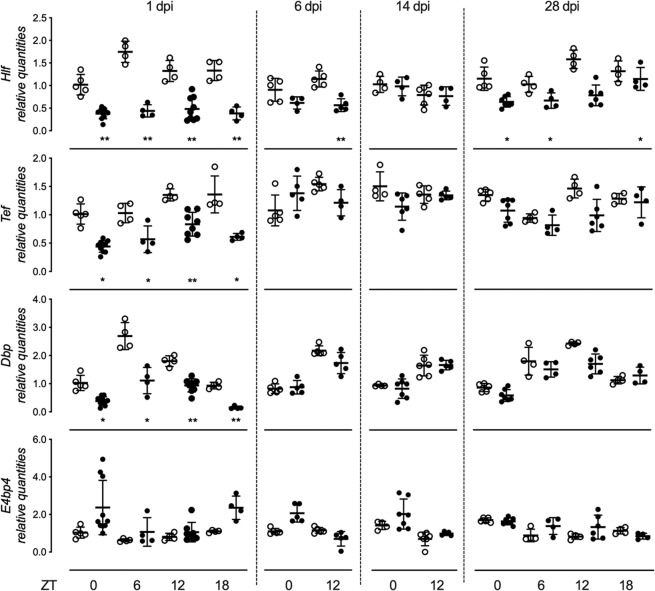
Relative mean expression (±SD) of PAR bZIP TFs (*Hlf*, *Tef*, *Dbp* and *E4bp4*) in contralateral (non-injected) dorsal hippocampus at 1, 6, 14 and 28 days post injection (dpi) of KA in adult male mice. ZT0, ZT6, ZT12 and ZT18 (ZT = Zeitgeber time, ZT0 lights on, ZT12 lights off). White and black circles represent individual values for control and KA groups, respectively. The t-test with multiple-testing correction was performed to evaluate significance between control and KA groups. *p < 0.05, **p < 0.005. Figures were created using GraphPad Prism version 8.3.1 for macOS, www.graphpad.com.

**Figure 4:**
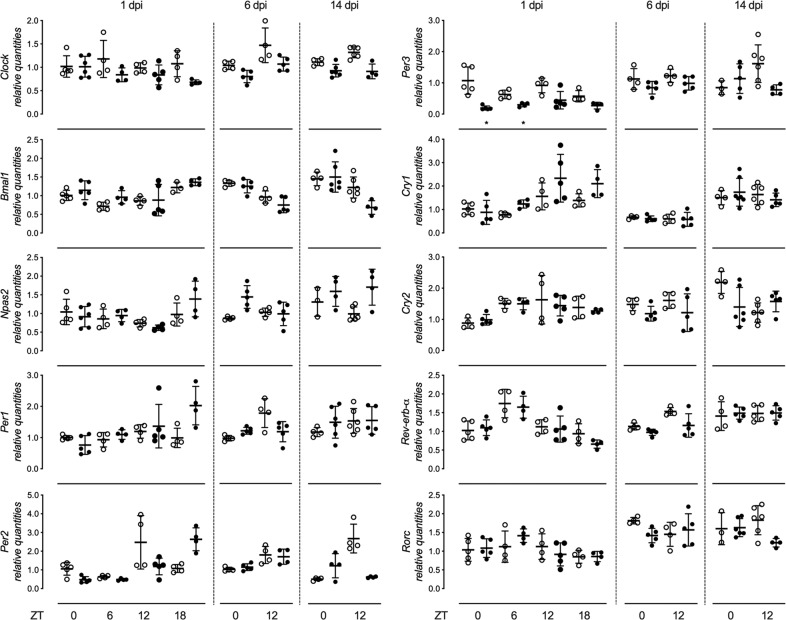
Relative mean expression (±SD) of clock genes (*Clock, Bmal1, Npas2, Per1, Per2, Per3, Cry1, Cry2, Rev-erb-α* and *Rorc*) in ipsilateral dorsal hippocampus at 1, 6 and 14 days post injection (dpi) of KA in adult male mice. ZT0, ZT6, ZT12 and ZT18 (ZT = Zeitgeber time, ZT0 lights on, ZT12 lights off). White and black circles represent individual values for control and KA groups, respectively. The t-test with multiple-testing correction was performed to evaluate significance between control and KA groups. *p < 0.05. Figures were created using GraphPad Prism version 8.3.1 for macOS, www.graphpad.com.

We evaluated the hippocampal expression of core clock genes and PAR bZIP TFs at different stages during epileptogenesis by injecting KA into the right dorsal hippocampus, a well-established model of TLE^35^. The pathogenesis of this model follows a stereotypic pattern^23,35^, characterized by an initial status epilepticus lasting up to 1 dpi, followed by a silent latent period and the occurrence of spontaneous recurrent seizures in the chronic period starting at about 14 dpi. The expression of clock genes and PAR bZIP TFs was evaluated at two (6 and 14 dpi) or four ZT time-points (1 and 28 dpi) over a day to exclude possible phase shift in clock gene expression amplitude. We found significant alteration in the expression of PAR bZIP TFs (Fig. 2). The transcriptional activators *Hlf*, *Dbp* and *Tef* were significantly down-regulated while the transcriptional repressor *E4bp4* was up-regulated. Changes were most evident during the acute and epileptogenesis phases (1 and 6 dpi, respectively) of the status-epilepticus and tend to normalize thereafter. The expression of *Hlf* remained significantly down-regulated also at the beginning of the chronic phase, 14 days after the induction of status epilepticus. At 28 dpi, *Hlf* was significantly suppressed only at ZT18. Interestingly, similar changes in expression of PAR bZIP TFs were found in the contralateral (not-injected) hippocampus at 24 h post injection. Additionally, *Hlf* remained significantly downregulated during the later chronic period (28 dpi) in the contralateral hippocampus (Fig. 3). Surprisingly, we did not observe any significant changes in the expression of the following clock genes: *Clock*, *Bmal1*, *Npas2*, *Per1*, *Per2*, *Cry1*, *Cry2*, *Rev-erb-α* and *Rorc* (Fig. 4). Only the expression of *Per3* was downregulated 24 h after the induction of status epilepticus (Fig. 4). In the contralateral hippocampus, there were no significant changes in the expression of core clock genes except downregulation of *Per3* at 1 dpi, ZT0 and 6 (see Supplementary Fig. 1). To ensure that the observed changes are due to the local KA injection, we analyzed in addition the expression of PAR bZIP TFs and core clock genes in the cortical tissue above the injected hippocampus. We did not detect any significant changes in this tissue (see Supplementary Fig. 2).

**Figure 5:**
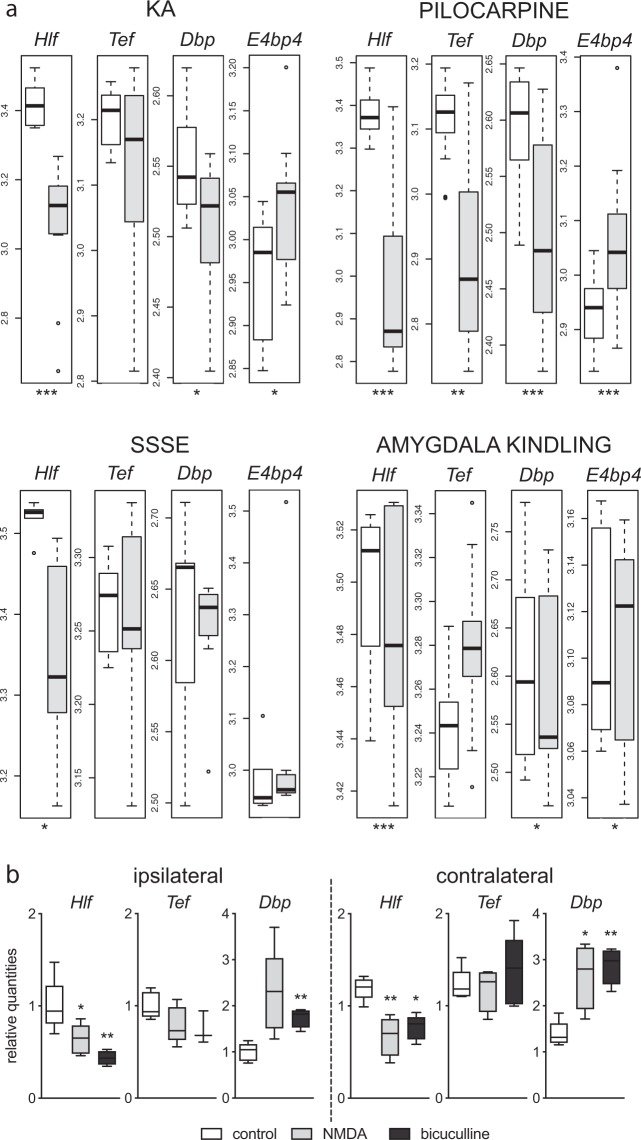
The expression of PAR bZIP TFs in other models of acquired epilepsy. (**a**) Hippocampal expression of PAR bZIP transcription factors (*Hlf, Dbp, Tef* and *E4bp4*) in four different rat models of status epilepticus: systemic KA (n = 12 in each group) and pilocarpine administration (n = 12 in each group), self-sustained status epilepticus (SSSE, n = 5 in control group, n = 6 in SSSE group) and amygdala kindling (n = 12 in each group)^26^. All rats involved in the study were male. White and grey boxes represent control and epilepsy model groups, respectively. Differential expression of the genes *Hlf*, *Tef*, *Dbp* and *E4bp4* was performed using the Linear models for microarray (Limma) (**b**) Relative expression of PAR bZIP TFs after intrahippocampal injection of NMDA or bicuculline in adult male mice (n = 5 in each group). The t-test was performed to evaluate significance. *p < 0.05, **p < 0.005, ***p < 0.001. Figures were created using R version 3.4.3., https://www.R-project.org and GraphPad Prism version 8.3.1 for macOS, www.graphpad.com.

To evaluate whether changes in PAR bZIP TFs can be observed in other rodent models of TLE, we performed analysis on previously published^26^ microarray data (NCBI Gene Expression Omnibus. GSE47752) profiling the hippocampal expression of genes in four different rat models of acquired epilepsy: kainate (intraperitoneal, i.p.), pilocarpine, self-sustained status epilepticus (SSSE) and amygdala kindling. We focused our analysis on the differential expression of PAR bZIP factors in these models at 24 h after induction of status epilepticus. Our analysis revealed signification downregulation of *Hlf* in all four models (See Fig. 5a). In addition, *Tef* was significantly downregulated and the transcriptional repressor *E4bp4* was significantly up-regulated in all models except for SSSE. *Dbp* was significantly downregulated in the pilocarpine model. Taken together the microarray data of four rat models of epilepsy support our finding of altered expression of PAR bZIP TFs in KA induced epilepsy.

### Excitotoxicity and seizure activity induce the downregulation of *Hlf*

Next, we investigated whether acute hyperexcitability in general is responsible for the altered behavior of PAR bZIP TFs. To explore this possibility, we injected either N-methyl-D-aspartic acid (NMDA) or bicuculline into the right dorsal hippocampus. Hippocampal injection of NMDA stimulates ionotropic NMDA receptors, induces acute excitotoxicity and status epilepticus; however, unlike KA-induced status epilepticus, does not cause an early loss of hippocampal interneurons^22,36^. Bicuculline is a competitive antagonist of GABA_A_ receptors and it is known to produce seizures in the absence of neurodegenerative events^37^. In both models, *Hlf* was significantly downregulated 24 h after status epilepticus (Fig. 5b), whereas *Dbp* was upregulated only after NMDA injection. These effects were mirrored also in the contralateral hippocampus (Fig. 5b).

### Changes in the expression of PAR bZIP transcription factors are independent of inflammation

**Figure 6:**
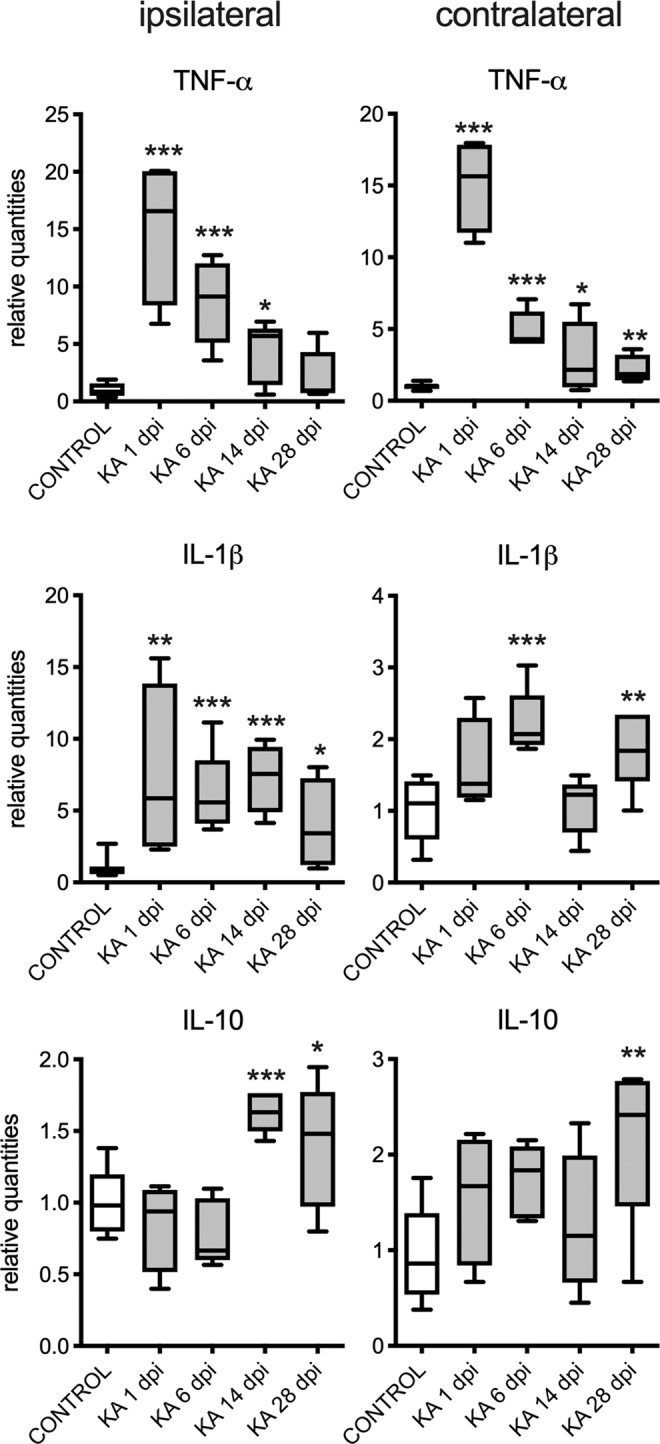
The relative expression of proinflammatory (TNF-α and IL-1β) and anti-inflammatory cytokines (IL-10) in ipsilateral and contralateral hippocampi at 1, 6, 14 and 28 days post injection (dpi) of KA in adult male mice (n = 5 in each group). The left panel shows changes in ipsilateral hippocampus. The right panel shows changes in contralateral hippocampus. The t-test with multiple-testing correction was performed to evaluate significance between control and KA groups *p < 0.05, **p < 0.005, ***p < 0.001. Figures were created using GraphPad Prism version 8.3.1 for macOS, www.graphpad.com.

Both clinical^38–40^ and experimental^20,41,42^ evidence suggest that inflammatory processes are involved in the pathogenesis of TLE. Hence, we characterized the expression of proinflammatory cytokines TNF-α and IL-1β, as well as the anti-inflammatory cytokine interleukin 10 (IL-10) in the intrahippocampal KA model of TLE (Fig. 6). TNF-α expression was significantly upregulated 1, 6 and 14 days after the KA infusion (15-fold, 10-fold and 5-fold respectively). IL-1β beta remained upregulated at all timepoints studied, the effect being about 5-fold. The expression of IL-10 was increased at later time points, at 14 and 28 dpi. These cytokines were also significantly upregulated in the contralateral hemisphere, albeit the increase being less pronounced (Fig. 6).

**Figure 7:**
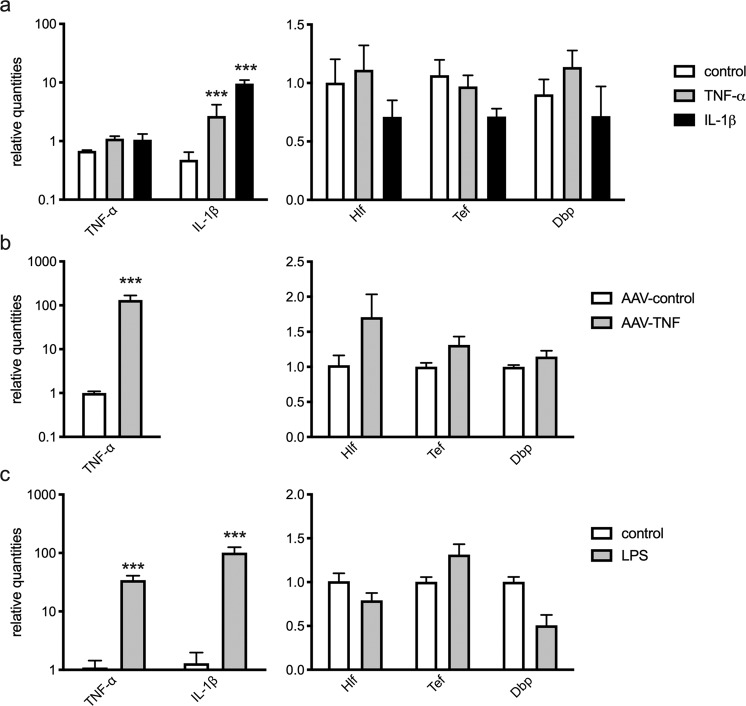
The effect of proinflammatory environment on the expression of PAR bZIP TFs in dorsal hippocampus of adult male mice (n = 5 in each group). Relative mean expression (±SD) of inflammatory cytokines and PAR bZIP TFs after (**a**) acute injection of mouse recombinant cytokines (TNF-α or IL1-β, 6 h after injection) (**b**) chronic overexpression of TNF-α (**c**) acute inflammation induced by injection of LPS (24 h after injection). The t-test with multiple-testing correction was performed to evaluate significance. ***p < 0.001. Figures were created using GraphPad Prism version 8.3.1 for macOS, www.graphpad.com.

As outlined above, inflammation regulates the expression of PAR bZIP TFs. Thus, we hypothesized that the KA induced inflammatory response drives the observed changes of PAR bZIP TFs expression. To test this hypothesis, we infused either mouse cytokine TNF-α or IL-1β into the hippocampus. Whereas TNF-α mRNA expression did not increase upon TNF-α or IL-1β injection, the expression of IL-1β mRNA was augmented about 5- and 10-fold respectively (Fig. 7a). However, we observed no changes in the expression of PAR bZIP factors (Fig. 7a). As cytokine levels reached by this treatment may have been insufficient to inhibit PAR bZIP TFs, we decided to simulate sub-chronic TNF-α exposure by infusion of an adenovirus-associated viral TNF vector (AAV-TNF). The AAV-TNF induced robust TNF-α expression (100- fold) in the hippocampus; however, it did not affect the expression of PAR bZIP TFs (Fig. 7b). We corroborated this result by inducing neuroinflammation by infusion of lipopolysaccharide (LPS). LPS activates microglia interacting with their Toll-like receptor-4 and induces production of inflammatory cytokines. The intrahippocampal infusion of LPS induced significant overexpression of TNF-α (50-fold) and IL-1β (100-fold); however, the expression of PAR bZIP TFs remained unchanged (Fig. 7c). Surprisingly, the hippocampal expression of PAR bZIP TFs was not affected by a local increase in the expression of pro-inflammatory cytokines such as TNF-α and IL-1β, as has been described for other murine tissues^15^. This evidence is in line with our previous finding that the expression of PAR bZIP TFs is unchanged in brains of mice with experimental autoimmune encephalomyelitis, a model of multiple sclerosis (unpublished data). Hence, this evidence suggests that early changes in hippocampal expression of PAR bZIP TFs in rodent models of TLE are not a direct response to inflammatory processes that occur during the initial stages of epileptogenesis. Thus, PAR bZIP TFs in the hippocampal tissues are regulated in a different way than in the periphery.

### KA induces early response of clock genes

**Figure 8:**
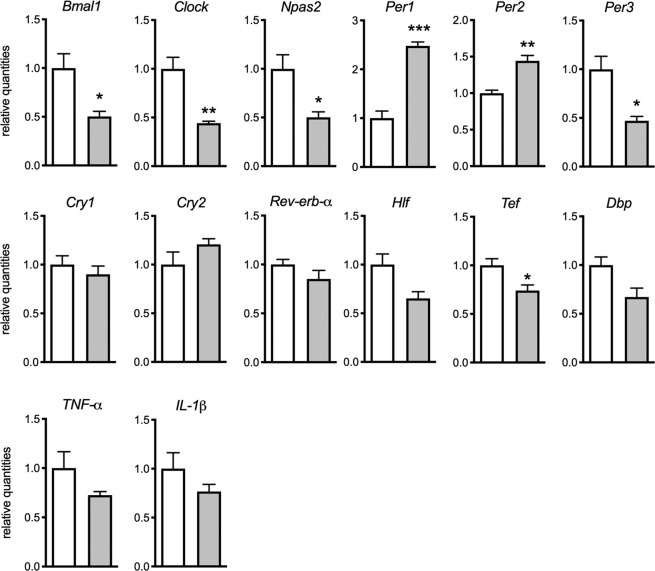
Relative mean expression (±SD) of clock genes, PAR bZIP TFs and proinflammatory cytokines at three hours after the injection of KA in adult male mice (n = 5 in each group). White and grey boxes represent control and KA groups, respectively. The t-test with multiple-testing correction was performed to evaluate significance. *p < 0.05, **p < 0.005, ***p < 0.001. Figures were created using GraphPad Prism version 8.3.1 for macOS, www.graphpad.com.

To further explore the mechanism responsible for the downregulation of PAR bZIP TFs during epileptogenesis, we decided to characterize the hippocampal expression of PAR bZIP TFs and core clock genes, at 3 hours after the induction of status epilepticus by intrahippocampal KA (Fig. 8). At this early time point, we observed significant reduction of *Clock, Bmal1, Npas2* and *Per3* expression. Additionally, the expression of *Per1* and *Per2* was significantly upregulated. The expression of PAR bZIP TFs *Hlf* and *Dbp* did not show statistically significant decrease, while the expression of *Tef* was significantly downregulated. This expression pattern suggests that early changes in core clock genes might be involved in the initiation of changes in PAR bZIP TFs levels. The positive regulators of their expression (*Clock, Bmal1, Npas2*) were downregulated and their negative regulators (*Per1* and *Per2*) were upregulated. Additionally, the expression of *TNF-α* and *IL-1β* was not altered yet at this early time point. These results might explain the changes in PAR bZIP TFs expression at 1 dpi after the KA lesion.

### Neurons over-expressing *Hlf* showed a significant decrease in the frequency and increase in the amplitude of spontaneous events in the presence of bicuculline and glutamate

**Figure 9:**
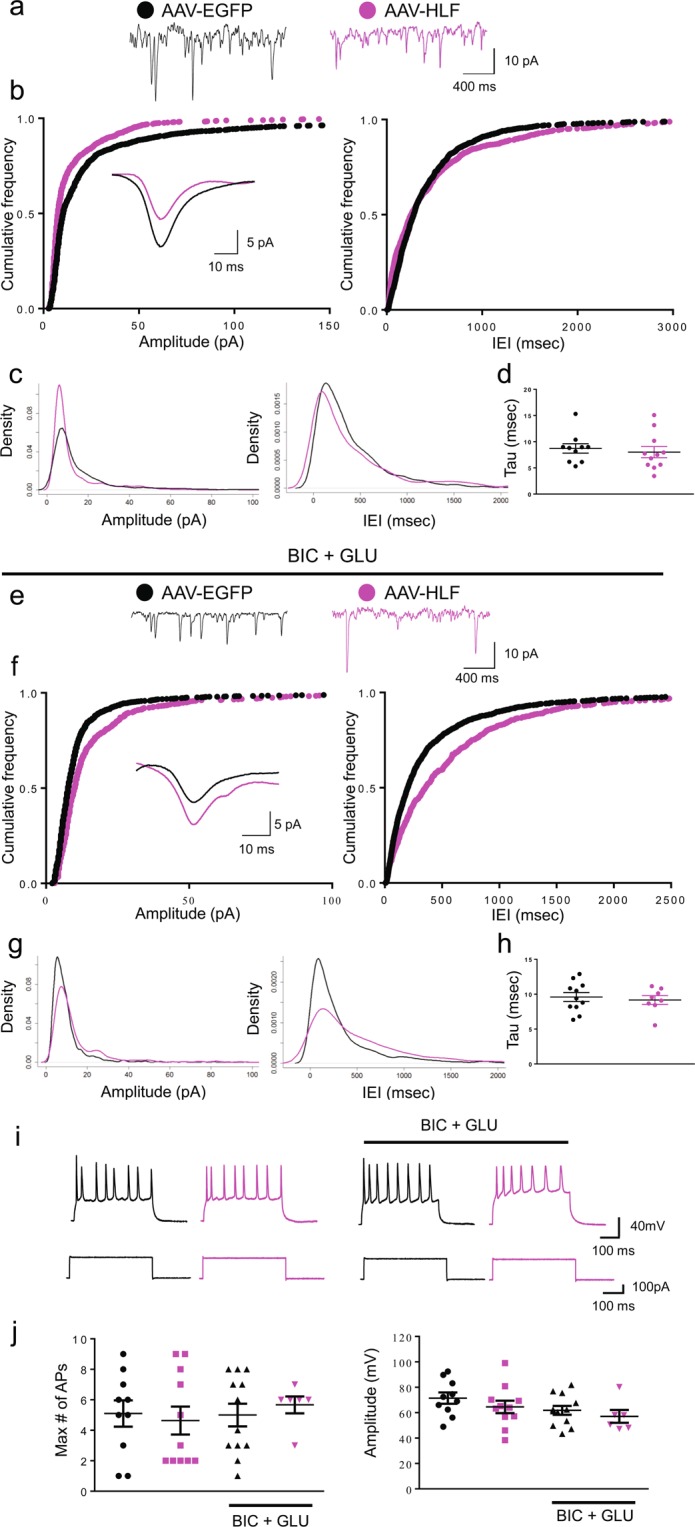
(**a–c**) *Hlf* overexpression decreases the amplitude but does not affect the frequency of spontaneous currents under basal conditions. (**a)** Representative traces (sPSCs). (**b)** (left) Cumulative amplitude distributions for sPSCs. 64.3% of simulated samples had a p-value < 5%. Inset: Mean spontaneous currents. (right) Cumulative interevent interval distributions for sPSCs. 20.7% of simulated samples had a p-value < 0.05. (**c)** Density plot of sPSCs (left) amplitudes and (right) interevent intervals. (**d)** Decay kinetics of sPSCs. (**e,f**) Hlf overexpression reduces the frequency of spontaneous excitatory currents in the presence of bicuculline and glutamate (BIC+GLU). (**e**) Representative sPSCs traces. (**f**) (left) Cumulative amplitude distributions for sPSCs. 67.6% of simulated samples had a p-value < 0.05. (right) Cumulative interevent interval distributions for sPSCs. 72.6% of the simulated samples had a p-value < 0.05. Inset: Mean spontaneous currents. (**g**) Density plot of sPSCs (left) amplitudes and (right) interevent intervals. (**h**) No significant differences were observed in decay kinetics between groups (**i,j**) There are no differences in action potential number between neurons transduced with AAV-EGFP or a AAV-HLF in basal conditions or in the presence of bicuculline and glutamate. (**i**) representative action potential (AP) traces fired by a current step of 250 nA in (left) basal conditions and (right) in the presence of BIC+GLU. (**j**) Left panel: AP number. Right panel: AP amplitude. AAV-EGFP: black traces and dots. AAV-HLF: magenta traces and dots. Figures were created using Adobe Illustrator version 23.1.1., www.adobe.com/Illustrator; R version 3.4.3., https://www.R-project.org; Mini Analysis version 6.0.7., http://www.synaptosoft.com/MiniAnalysis/; Pclamp suite version 10, https://www.moleculardevices.com/products/axon-patch-clamp-system/acquisition-and-analysis-software/pclamp-software-suite#gref and GraphPad Prism version 8.3.1 for macOS, www.graphpad.com.

As we observed consistent downregulation of *Hlf* expression in various models of epilepsy, likely as a result of acute hyperexcitability, we examined the effects of *Hlf* expression in neurons under basal and hyperexcitable conditions. Hence, we overexpressed *Hlf* by transducing primary hippocampal neurons at 2 DIV with either a control vector (AAV-EGFP) or a vector overexpressing *Hlf* (AAV-HLF) and recorded spontaneous currents at ~12–16 DIV by whole-cell voltage clamp. Under basal activity levels (Fig. 9a,b,c), overexpression of *Hlf* resulted in a significant reduction in the amplitude of sPSCs, with no differences in the frequency or decay kinetics (Fig. 9d, AAV-EGGP: n = 10, 8.72 ± 0.89 msec; AAV-HLF: n = 11, 8.01 ± 1.06 msec, p = 0.6204), of events compared to neurons transduced with an AAV-EGFP. In the presence of bicuculline and pressure-ejected glutamate (Fig. 9e,f,g), neurons overexpressing *Hlf* showed a significant decrease in the frequency and an increase in the amplitude of sEPSCs compared to neurons carrying the EV control. We observed no differences between groups in the number of action potentials [AAV-EGFP: n = 10, 5.1 ± 0.86, AAV-HLF: n = 11, 4.64 ± 0.92, AAV-EGFP (BIC + GLU): n = 12, 5 ± 0.75, AAV-HLF/(BIC + GLU): n = 6, 5.67 ± 0.56. ANOVA: F (3, 35) = 0.2016, P = 0.8946)] or amplitude [AAV-EGFP: n = 10, 71.49 ± 4.45 mV AAV-HLF: n = 11, 64.57 ± 4.92 mV, AAV-EGFP/(BIC + GLU): n = 12, 61.83 ± 3.51 mV, AAV-HLF/(BIC+GLU): n = 6, 57.08 ± 5.03 mV ANOVA: F (3, 35) = 1.545, P = 0.2200)]; Fig. 9i,j.

### Gene expression analysis

To determine which genes are regulated by hepatic leukemia factor, we performed transcriptome analysis of the dorsal hippocampal tissue from mice over-expressing mouse *Hlf* restricted to the *dentate gyrus* (Fig. 1a). It has been shown that that mouse endogenous *Hlf* is expressed mostly in the dentate gyrus, while in *CA1* and *CA3* subregions of hippocampus the expression is limited^43^, (Experiment: 565 and 566, Probe Name: Rp_Baylor_103366)^44^. These samples were compared with samples from mice injected with a control vector (AAV-EGFP). *Hlf* was over-expressed 7-fold in the dorsal hippocampal tissue while there were no changes in the expression of *Tef* and *Dbp* (Fig. 1b). In addition, the AAV-HLF injection did not induce local neuroinflammation detected as expression of TNF-α and IL-1β (Fig. 1b). The expression of AAV-HLF vector was restricted to neurons and was detected in neither astrocytes nor microglia (Fig. 1c). Only samples with confirmed overexpression of *Hlf* using qPCR were sequenced (AAV-HLF n = 7, AAV-EGFP n = 7). In total, the expression of 65 genes was significantly altered (Fig. 1d). The list of all differentially expressed genes is included in the Supplementary Table S1. Raw data are available at NCBI GEO database under accession number GSE140046. Additionally, the gene ontology classification for molecular function and biological process were performed using all 65 significantly differentially expressed genes (Fig. 1e,f).

We identified genes whose expression is influenced by neuronal *Hlf*. We found changes in expression of genes coding for ion channels such as *Trpa1*, *P2rx5*, *Grin3a*, *Kcng1* and *Gabrd*. Next, the expression of *Synpr*, *Gfra2*, *Lcn2*, *Slc30a3*, *Rasd2*, *Igfbp5*, *Fxyd7*, *Cdkn1a* and *Slc6a8* was altered. Synaptoporin, the protein coded by *Synpr* gene, is a marker of mossy fiber sprouting, a phenomenon associated with epileptogenesis after KA injection^45^. *Gfra2* was reported to modulate threshold of kindling evoked seizures^46^, *Lcn2* was identified as a chemokine inducer in KA model^47^ and *Slc30a3* modulates transport of zinc into synaptic vesicles that is co-released with glutamate and regulates excitability and its loss is associated with febrile seizures^48^. *Rasd2* and *Igfbp5* were identified by a recent study as candidate genes associated with epileptogenicity in KA model of TLE^49^ and *Fxyd7* to be altered after seizure preconditioning using pilocarpine model^50^. Human studies reported *Cdkn1a* to be upregulated in patients with epilepsy^51^ and deficiency in *Slc6a8* was reported to result in intractable epilepsy and cognitive impairment^52^. Although, with the given dataset, we are not able to deduce any direct involvement in seizure modulation, many of those genes were previously described to be associated with neuronal excitability, seizures or epilepsy.

## Discussion

Even though the occurrence of epileptic seizures in circadian patterns has been documented in animal models as well as humans^53^ the relationship between epileptic seizures and the circadian system at the molecular level is still unclear. A recent study reported decreased *Clock* expression in human epileptogenic tissue and decreased seizure threshold in mice with *Clock* deletion in excitatory cortical neurons suggesting that alterations in *Clock* expression are epileptogenic^3^. Furthermore, mice deficient for *Bmal1* exhibited reduced seizure thresholds^13^.

In this study, we utilized the KA model of TLE to study the expression of core clock genes and their downstream transcription factors, the PAR bZIP TFs family. We showed that the expression of *Hlf*, *Dbp*, *Tef* is suppressed while the expression of *E4bp4* is significantly upregulated during epileptogenesis. This effect was most evident during the acute phase (1 dpi); however, it was significant also during epileptogenesis (6 dpi) and at the beginning of the chronic phase (14 dpi). Additionally, our analysis of existing microarray data set (Dingledine R. 2013. NCBI Gene Expression Omnibus. GSE47752) revealed identical trends in the expression of PAR bZIP TFs in rat models of acquired epilepsy. Surprisingly, the expression of core clock-genes was not altered at 1, 6, 14 or 28 dpi after KA injection. These data are in accordance with another study performing gene profiling in dorsal hippocampus after 6 hours, 15 days and 6 months post KA injection^54^. They did not find significant changes in core-clock gene expression while they identified *Hlf* to be an important transcription factor associated with changes in gene expression at 15 dpi. The functional importance of PAR bZIP TFs in regulating neuronal excitability was proven by a study showing that triple-knockout mice deficient for *Hlf*, *Dbp* and *Tef* develop audiogenic and spontaneous seizures^12^. The authors pointed at *Tef* as the gene associated with seizures by regulating expression of pyridoxal kinase, an enzyme involved in the metabolism of neurotransmitters. In another study, the deletion of *Hlf* in mouse exacerbated seizures and reduced survival in the Snc2a^Q54^ mouse model of epilepsy^55^. Likewise, DBP was found downregulated together with CLOCK, CRY1 and PER1 at protein levels in patients with focal cortical dysplasia^3^. Furthermore, neuroprotective effects were described for *E4bp4*, which we found here to be upregulated after induction of status epilepticus. Besides modulating circadian rhythms, *E4bp4* serves as a survival factor in motoneurons during their development^56^. By limiting neuronal injury, the upregulation of *E4bp4* in experimental epilepsy may be a part of the response to neurotoxic insults.

Interestingly, KA induced changes in the expression of PAR bZIP TFs as well as neuroinflammation were mirrored in the contralateral (non-injected) hippocampus. *Hlf* remained downregulated there even at the late chronic phase (28dpi). At the acute phase, this effect is most likely attributed to hyperactivity spreading from the injected hippocampus via commissural projections^57^. However, during the chronic phase the epileptic activity does not spread into the contralateral hippocampus^58^. Additionally, it has been shown that contralateral hippocampus can provide important regulatory input and modulate ongoing seizures^59^. Thus, it is not clear whether the downregulation of *Hlf* in contralateral hippocampus during the chronic phase is caused by ongoing changes in the ipsilateral hippocampus or it is entrained by intrinsic changes in the contralateral hippocampus induced during the status epilepticus.

What induces the changes in PAR bZIP TFs expression? We have shown in our previous research that the expression of PAR bZIP TFs is suppressed by the proinflammatory cytokines TNF-α and IL-1β by interfering with their E-boxes in mouse liver and fibroblasts^15^. Besides suppressing *Hlf*, *Dbp* and *Tef*, TNF-α was found to enhance the expression of *E4bp4* in synovial fibroblasts^18^. This picture is identical to the one detected here in our KA induced model of epilepsy. Since we observed an elevated expression of TNF-α and IL-1β in the KA model, we thought that the cytokine induced neuroinflammatory effect might be responsible for the modulation of PAR bZIP TFs. However, we were not able to suppress the expression of PAR bZIP TFs in hippocampus by simulating the proinflammatory environment. This suggests that their expression in the hippocampus is regulated by mechanisms than differ from those in periphery. Distinct circadian oscillations of PAR bZIP TFs in different tissues supports this hypothesis. In liver and kidney, PAR bZIP TFs oscillate diurnally with a high amplitude^60,61^ while in brain tissue they oscillate with a low amplitude or not at all^11,12^.

Early changes in clock core-genes expression might be driving the initial downregulation of PAR bZIP TFs. At an early time-point (3 h) after KA injection, when the inflammatory cytokines are not yet elevated, the expression of *Clock*, *Npas2* and *Bmal1* were significantly downregulated while *Per* genes 1 and 2 were upregulated. The expression of *Dbp* and presumably also *Hlf* and *Tef* is directly activated by CLOCK-BMAL1^62,63^ and inhibited by PER and CRY^64^, thus their deficient expression results in altered expression of PAR bZIP TFs. In addition, it has been shown that *Hlf* and *Tef* expression in neurons is reduced in mouse with neuronal *Clock* deletion^3^. However, it is unclear whether these early changes in core clock gene expression contribute to alterations in PAR bZIP TFs at later stages of KA model, since their expression is already normalized at this stage. Alternatively, the neuroinflammation, excitotoxicity or seizure activity itself might play a role in sustaining the altered levels of PAR bZIP TFs.

To further investigate the role of PAR bZIP TFs in the regulation of neuronal excitability, we induced *Hlf* over-expression in primary neurons under hyperexcitable conditions induced by glutamate and bicuculline exposure. It resulted in decrease in the frequency and an increase in the amplitude of sEPSCs. In another study, *Hlf* deletion has been shown to increase the frequency of spontaneous seizures in two mouse models involving mutations on voltage-gated sodium channels; the gain-of-function Scn2a^Q54^ (Q54) mouse model and the heterozygous loss-of-function Scn1a mouse model^55^. Interestingly, these models seem to induce seizures from different mechanisms; a reduction in GABAergic inhibition in the Scn1a model and an increase in excitability in CA1 glutamatergic neurons in the Q54 mouse model^65^. This posits the possibility that *Hlf* is involved in regulation of both pathways, which may explain the lower excitability we observed when exposing hippocampal primary neurons to hyperexcitable conditions. This decrease in excitability does not appear to involve changes in intrinsic properties of neurons nor in dendritic filtering, as we did not observe changes in the number or amplitude of action potentials, nor in the decay time of spontaneous currents. This result suggests that *Hlf*, as a member of PAR bZIP TFs, is an intrinsic regulatory element modulating synaptic activity by a yet unknown mechanism.

Taken together, *Hlf* seems to be a promising transcription factor associated with epileptogenesis. We found consistent deficiency of *Hlf* in animal models of acquired epilepsy, demonstrated that *Hlf* regulates neuronal activity and its overexpression in neurons leads to altered expression of genes associated with epilepsy. Upon resolving the molecular mechanism and causality between PAR bZIP TFs alterations and seizures, these findings should initiate further studies into PAR bZIP TFs as a target to prevent epileptogenesis or to modulate seizure activity.

## Supplementary information

Supplementary Data.

Supplementary Data1.

Supplementary Data2.

Supplementary Data3.
