# Supplementary material for: Aberrant expression of PAR bZIP transcription factors is associated with epileptogenesis, focus on hepatic leukemia factor
Source: Sci Rep. 2020 Feb 28;10:3760. doi: 10.1038/s41598-020-60638-7 (PMC7048777; doi:10.1038/s41598-020-60638-7)
Supplement: Supplementary file 1 — Supplementary Data. [file 41598_2020_60638_MOESM1_ESM.docx]

**Aberrant expression of PAR bZIP transcription factors is associated with epileptogenesis, focus on hepatic leukemia factor**

Rambousek Lukas^1^*, Gschwind Tilo^2,3^, Lafourcade Carlos^4^, Paterna Jean-Charles^5^, Dib Linda^6^, Fritschy Jean-Marc^2,3^ and Fontana Adriano^1^

1 Institute of Experimental Immunology, Winterthurerstrasse 190, University of Zurich, 8057 Zurich, Switzerland

2 Institute of Pharmacology and Toxicology, Winterthurerstrasse 190, University of Zurich, 8057 Zurich, Switzerland

3 Neuroscience Center Zurich, University of Zurich and ETH Zurich, 8057 Zurich,

Switzerland

4 Laboratorio de Neurociencias, Universidad de los Andes, 12455 Santiago, Chile

5 Viral Vector Facility, Neuroscience Center Zurich, University of Zurich and ETH Zurich, 8057, Zurich, Switzerland.

6 Swiss Institute of Bioinformatics, 1015 Lausanne, Switzerland

**Supplementary Information File**

**Supplementary Figure 1**

Relative mean expression (± SD) of clock genes (*Clock, Bmal1, Npas2, Per1, Per2, Per3, Cry1, Cry2, Rev-erb-α* and *Rorc*) in contralateral (non-injected) dorsal hippocampus at 1, 6 and 14 days post injection (dpi) of KA in adult male mice. ZT0, ZT6, ZT12 and ZT18 (ZT = Zeitgeber time, ZT0 lights on, ZT12 lights off). White and black circles represent individual values for control and KA groups, respectively. The t-test with multiple-testing correction was performed to evaluate significance between control and KA groups. * p < 0.05. Figures were created using GraphPad Prism version 8.3.1 for macOS, [www.graphpad.com](http://www.graphpad.com).

**Supplementary Figure 2**

Relative mean expression (± SD) of PAR bZIP TFs (*Hlf*, *Tef*, *Dbp* and *E4bp4*) and selected clock genes (*Clock, Bmal1, Per1, Per2, Per3, Cry1* and *Cry2,*) in cortical tissue overlying the ipsilateral dorsal hippocampus at 1, 6, 14 and 28 days post injection (dpi) of KA in adult male mice at ZT0 (ZT = Zeitgeber time, ZT0 lights on, ZT12 lights off). White and black circles represent individual values for control and KA groups, respectively. The t-test with multiple-testing correction was performed to evaluate significance between control and KA groups. * p < 0.05. Figures were created using GraphPad Prism version 8.3.1 for macOS, [www.graphpad.com](http://www.graphpad.com).
