# Supplementary figures and images for: Aberrant expression of PAR bZIP transcription factors is associated with epileptogenesis, focus on hepatic leukemia factor
Source: Sci Rep. 2020 Feb 28;10:3760. doi: 10.1038/s41598-020-60638-7 (PMC7048777; doi:10.1038/s41598-020-60638-7)

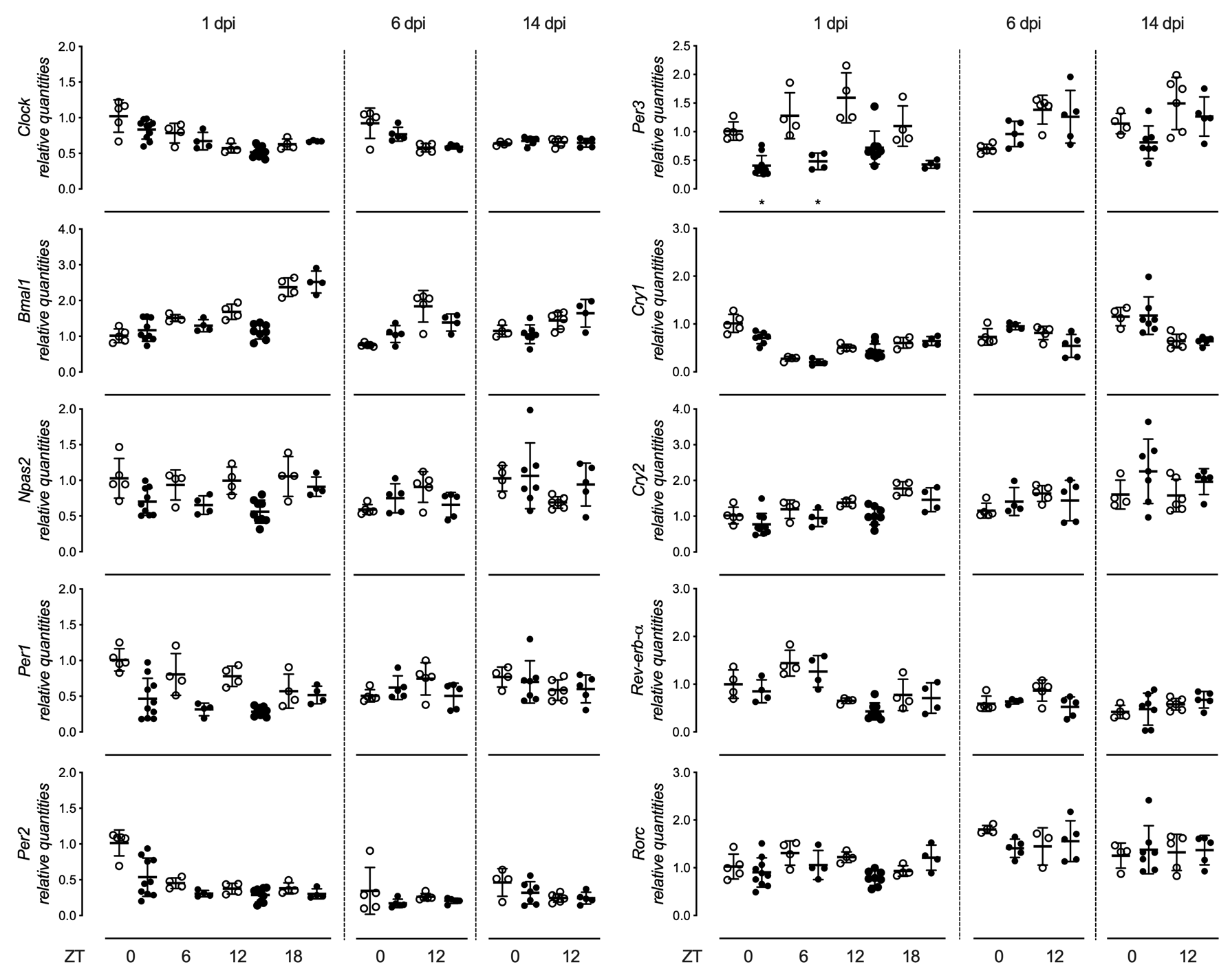

Supplement: Supplementary file 3 — Supplementary Data2. [file 41598_2020_60638_MOESM3_ESM.tiff]

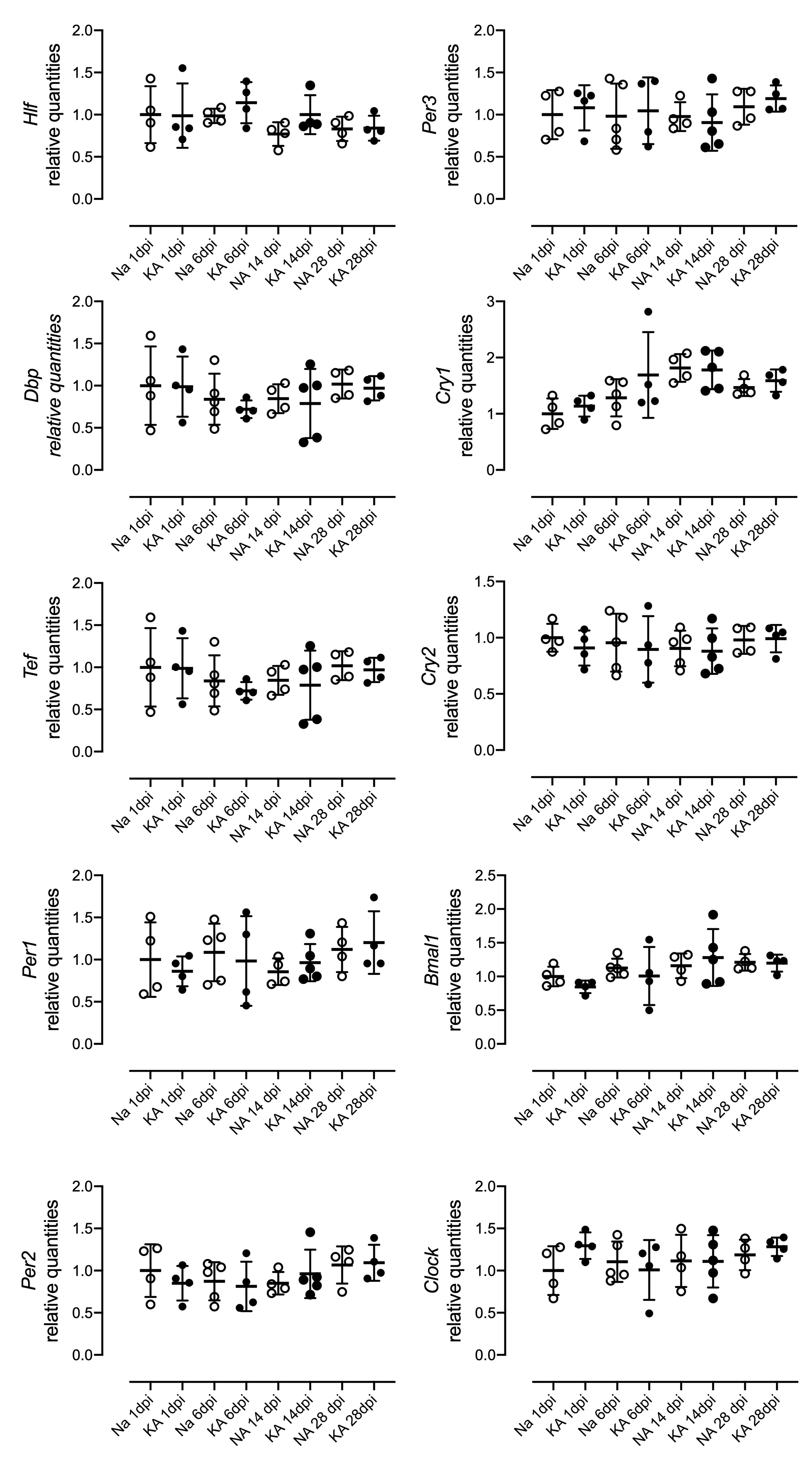

Supplement: Supplementary file 4 — Supplementary Data3. [file 41598_2020_60638_MOESM4_ESM.tiff]
